# The impact of household wealth on child survival in Ghana

**DOI:** 10.1186/s41043-016-0074-9

**Published:** 2016-11-22

**Authors:** Stella T. Lartey, Rasheda Khanam, Shingo Takahashi

**Affiliations:** 1 Ministry of Health, Accra, Ghana; 2 School of Commerce, Faculty of Business, Education, Law and Arts, University of Southern Queensland, Toowoomba, Queensland 4350 Australia; 3 International University of Japan, Minami Uonuma, Japan

**Keywords:** Child survival, Household wealth, Weibull hazard model, Gamma frailty, Ghana

## Abstract

**Background:**

Improving child health is one of the major policy agendas for most of the governments, especially in the developing countries. These governments have been implementing various strategies such as improving healthcare financing, improving access to health, increasing educational level, and income level of the household to improve child health. Despite all these efforts, under-five and infant mortality rates remain high in many developing nations. Some previous studies examined how economic development or household’s economic condition contributes to child survival in developing countries. In Ghana, the question as to what extent does economic circumstances of households reduces infant and child mortality still remain largely unanswered. Thus, the purpose of this study is to investigate the extent to which wealth affects the survival of under-five children, using data from the Demographic and Health Survey (DHS) of Ghana.

**Methods:**

In this study, we use four waves of data from Demographic and Health Surveys (DHS) of Ghana from 1993 to 2008. The DHS is a detailed data set that provides comprehensive information on households and their demographic characteristics in Ghana. Data was obtained by distributing questionnaires to women (from 6000 households) of reproductive age between 15 and 49 years, which asked, among other things, their birth history information. The Weibull hazard model with gamma frailty was used to estimate wealth effect, as well as the trend of wealth effect on child’s survival probability.

**Results:**

We find that household wealth status has a significant effect on the child survival in Ghana. A child is more likely to survive when he/she is from a household with high wealth status. Among other factors, birth spacing and parental education were found to be highly significant to increase a child’s survival probability.

**Conclusions:**

Our findings offer plausible mechanisms for the association of household wealth and child survival. We therefore suggest that the Government of Ghana strengthens and sustains improved livelihood programs, which reduce poverty. They should also take further initiatives that will increase adult education and improve health knowledge. To the best of our knowledge, this is the first study in Ghana that combines four cross sectional data sets from DHS to study a policy-relevant question. We extend Standard Weibull hazard model into Weibull hazard model with gamma frailty, which gives us a more accurate estimation. Finally, the findings of this study are of interest not only because they provide insights into the determinants of child health in Ghana and other developing countries, but they also suggest policies beyond the scope of health.

## Background

Efforts to reduce preventable deaths in children under 5 remained one of the major premises for setting the third goal in the Sustainable Development Goals (SDGs); thus, the world is currently working towards achieving good health and well-being by 2030 [45]. Improving child health in the developing world was one of the major targets of national governments and international organizations during the operationalization of the Millennium Development Goals (MDGs), and countries were required to give definite account of their efforts to achieve the MDGs in 2015 [44].

Throughout the past two decades, a number of strategies were proposed and implemented in order to reduce child mortality and improve child health in developing nations. Some of these strategies include improving health care financing, improving access to healthcare, increasing educational level, and, most importantly, efforts to reduce poverty. Despite all these efforts, under-five and infant mortality rates remain high in many developing nations.

Among the strategies listed, economic development and poverty reduction are deemed as major strategies that affect child health outcomes. For example, Pritchett and Summers [38] found that more than half a million child deaths, which occurred in developing world in 1990 alone, could be attributed to poor economic performance in the 1980s. Thus, economic development could contribute to child survival in a major way. If the state of the economy were better, it would increase the average income of the population, which would then increase capital for further investments [6], and also improve infrastructure, which would then positively affect individuals in the population.

In Ghana, the question as to what extent does economic circumstances of households reduces infant and child mortality remains largely unanswered. Thus, the purpose of this study is to investigate the extent to which wealth affects the survival of under-five children, using data from the Demographic and Health Survey (DHS) of Ghana. We infer that households’ wealth reduces under-five mortality rate, since children from wealthier households may be exposed to less health shocks than children from poor backgrounds, given that rich parents are able to provide nutritious food, clean water and a safe environment (among other factors) for their children. In this sense, we expect the household wealth to be substitute to publicly offered child health care and public infrastructure in general. Given that public health care and infrastructure have improved in the decades, we also expect that the wealth effects might be reduced over time. Thus, we additionally investigate if the effects of wealth on under-five mortality have reduced overtime.

The notable studies that examined the relationship between child survival and mortality and household wealth in the developing countries are Chalasani and Rutstein (2012), Chalasani and Rutstein [14] and Schoeps et al. [43]. Using data from the Indian National Family Health Surveys, Chalasani and Rutstein [14] examined infant and under-five mortality and malnutrition outcomes. They found that the relationship between household wealth and under-five mortality reduced over time, especially for boys, while the relationship between malnutrition and household wealth became stronger for both boys and girls. By observing 1201 childhood deaths in rural and semi-urban Burkina Faso, Schoeps et al. [43] found that 5-year child survival probability is 93.6 and 88% in the semi-urban and rural area, respectively. Krishna et al. [32] investigated the associations between household wealth and physical growth of children using data from low- and middle-income countries and found that household wealth in early life matters for physical growth. Musafili et al. [35] investigated the trends and social differences in child mortality in Rwanda 1990–2000 and found that childhood mortality has decreased in Rwanda during this period and it has occurred due to reduction in social inequality.

Mostly due to data limitation, different studies, especially studies from developed countries, used “socioeconomic status” of the household to study this relationship. Most of these studies found a positive relationship between socioeconomic effect and child health [10, 11, 18, 29, 30]. The most common variable used as a proxy for socioeconomic status of the household in recent past has been maternal educational status [2, 9, 16, 17]. Unlike these studies, other studies have the perspective that data on income would give a better picture of socioeconomic effect on child mortality and survival [12, 38]. However, in the absence of income, consumption, or expenditure data, various studies have suggested that household assets and characteristics when weighted appropriately using the Principal Component Analysis could be used as proxy for the household wealth [13, 14, 21, 25, 39, 42].

This paper uses this strategy and makes several contributions to the literature. First, to the best of our knowledge, this is the first study in Ghana that combines four cross-sectional data sets from DHS to study a policy-relevant question. Further, we use principal component analysis (PCA) to measure wealth status of the households in the absence of data on income, consumption, and expenditure in DHS. As we use information on household’s assets and characteristics from four data sets from DHS for the years of 1993–2008 to construct wealth index; therefore, the wealth index we use serves as a long-term robust measure of household’s economic situation compared to income and expenditure.

Secondly, to obtain a more accurate estimation of the effect of wealth status on child survival, we extend Standard Weibull hazard model into Weibull hazard model with gamma frailty. Thus, unobserved heterogeneity and dependence among observations are two identification problems, which could lead to biased estimations in this study. In an attempt to account for these two, we include a gamma frailty term in our model [4, 23, 31, 36]; thus, the hazard function becomes a function of both the observed covariates and unobserved frailties associated with the individual. This is a unique contribution of this study.

Finally, the findings of this study are of interest not only because they provide insights into the determinants of child health in Ghana and other developing countries but they also suggest policies beyond the scope of health. This requires policy makers to collaborate with sectors outside of health in order to maximize the health of children.

## Methods

### Econometric model

Duration analysis was employed to determine the effect of wealth and other variables on the risk of death. Data used to examine this relationship is a cross section survey data with retrospective question on the state of children who are 5 years or below. In the DHS data set, we observe either the age of the child at the survey date or age of death, indicating that the data consist of both completed durations and right censored durations.

The major advantage of using this model is its ability to account for the sequential nature of the data; its ability to handle censoring and also its ability to incorporate time varying covariates. In this case, using a proportional hazard model makes it possible to estimate age pattern mortality. This is done through the estimation of hazard rate, which refers to the chances of making a transition from the current state at each instant conditioned on survival up to that point. The major difference between the various duration models is determined by the distribution that the function follows [Jenkins SP: Survival Analysis, unpublished].

It is widely believed that the conditional probability of a child’s survival increases as he/she progresses in age; thus, child survival is subject to “negative duration dependence”. Substantial policy interventions have been carried out in Ghana that promised an increase in child survival on the assumption that negative duration dependence is a pervasive phenomenon. This study estimates the duration dependence effects using the Weibull Hazard Model. The model adopted for this duration analysis is a flexible parameterization which is useful when the relationship we observe monotonically increases or decreases or it is flat with respect to time; it permits the baseline hazard to change with time, thus, capturing duration dependency.^1^ We estimate a simple child survival function that is a function of socioeconomic and proximate factors:

$$ \lambda \left({x}_i,\alpha, \beta, {\theta}_i\right)=\alpha {t}^{1-\alpha } \exp \left(x\hbox{'}\beta \right){\theta}_i $$

where *x*_*i*_ is a vector of socioeconomic and proximate determinants, respectively, for the *i*th child. Since we use a repeated cross section data, the covariates do not change with the survival time, and therefore, the covariates do not have the time subscript. By assuming that all the covariates are exogenous, we rule out other selective factors or policy initiatives, which improve, for example, the chances of survival of a child from a poor household. Thus, we use this to set an arbitrary external conditions, and in an attempt to account for the unobserved heterogeneity, the term, *θ*_*i*_, is used to represent unobserved heterogeneity, or frailty, associated with child survival which is assumed to be uncorrelated with the determinants in the survival function. We assume *θ*_*i*_, follows gamma distribution. Further explanation is given in Box-Steffensmeier and Jones [4].

### Data, variables, and summary statistics

#### Data description

The study uses data from the Demographic and Health Survey (DHS), which is the most detailed dataset on households and demographic characteristics in Ghana. It is a repeated cross-sectional data. The surveys collect information on a wide set of variables at the individual, household, and community levels and are conducted every 5 years. The sample for the survey covers about 6000 households in each round. Data was obtained by distributing questionnaires to women of reproductive age between 15 and 49 years, which asked, among other things, their birth history information. DHS dataset is divided into the following groups: birth, couple, household, individual, children, male, household member, verbal autopsy, and geographic datasets. We use the children dataset, which contains detailed child information as well as those of mother and the household.

In Ghana, there have been five rounds of collection, but only four rounds of datasets from 1993, 1998, 2003, and 2008 were used in this analysis; 1988 datasets were not used since some key variables, such as categorical regional data, were missing from it. There were 2204 observations in the 1993 wave, 3298 in the 1998 wave, 3844 in the 2003 wave, and 2992 observations in 2008 wave. After eliminating observations with incomplete information, our final sample contained 12,002 child year observations.

#### Variable description

Table 1 shows a description of the main variables used for our hazard function estimation. The selection of explanatory variables mostly follows prior literature, especially those suggested by Mosley and Chen [34]. Duration of survival for children was the main health indicator, which ranges between 0 and 59 months because the questionnaire asks about children whose ages were 5 years or less from the date of the interview.

**Table 1:** Description of variables used for analysis

Variables	Description
Duration	Age in months of the child at the time of survey. If the child is dead at the time of the survey, it shows the child’s age in month when the child died.
Household level	
Wealth index	Continuous variable which represents the long run economic status of household
Poorest	=1 if household is poorest quantile, 0 otherwise
Poor	=1 if household is poor quantile, 0 otherwise
Middle	=1 if household is middle quantile, 0 otherwise
Richer	=1 if household is richer quantile, 0 otherwise
Richest	=1 if household is richest quantile, 0 otherwise
Mothers’ age (years)	
Teenage mother	=1 if mother at the time of birth of the index child was 15 years and above but less than 20 years, 0 otherwise
20–29	=1 if mother at the time of birth of the index child was aged 20 or higher less than 30 years, 0 otherwise
30–39	=1 if mother at the time of birth of the index child was aged 30 or higher less than 40 years, 0 otherwise
Over 40	=1 if mother at the time of birth of the index child was age 40 or above, 0 otherwise
Mothers’ education	
No education	=1 if mother had never attended school, 0 otherwise
Primary	=1 if mother had primary education, 0 otherwise
Secondary or higher	=1 if mother had either secondary or higher education, 0 otherwise
Fathers’ education	
No education	=1 if father had never attended school, 0 otherwise
Primary	=1 if father had primary education, 0 otherwise
Secondary or higher	=1 if father had either secondary or higher education, 0 otherwise
Improved water	=1 if household’s source of drinking water is approved by WHO/UNICEF as improved, 0 otherwise
Improved sanitation	=1 if household uses toilet facility approved by WHO/UNICEF as improved, 0 otherwise
Individual level	
Male	=1 if sex of child is male, 0 otherwise
Birth order	Indicates the order in which index child was born
Preceding birth interval (months)	Indicate the difference in months between the index child and previous child
Below 24	=1 if preceding birth interval is less than 24 months, 0 otherwise
24–36	=1 if preceding birth interval is between 24–36 months, 0 otherwise
Above 36	=1 if preceding birth interval is above 36 months, 0 otherwise
Number of Children aged ≤5	Indicates the number of children in the household who are 5 years and below
Twin	=1 if child was of multiple birth, 0 otherwise
Community level	
Urban	=1if location was classified as urban, 0 otherwise
Regional distribution	
Southern Belt	=1 if household is located in Central or Western or Greater Accra Region, 0 otherwise
Eastern-Volta	=1 if household is located in Eastern or Volta Region, 0 otherwise
Ashanti-Brong	=1 if household is located in Ashanti or Brong-Ahafo Region, 0 otherwise
Northern Belt	=1 if household is located in Northern or Upper-East or Upper-West Region, 0 otherwise
Religion	
No religion	=1 if mother did not join any religious group, 0 otherwise
Christianity	=1 if mother was a Christian, 0 otherwise
Muslim	=1 if mother was a Muslim, 0 otherwise
Traditional	=1 if mother joins any Traditional religious sect, 0 otherwise
Others	=1 if mother joins any other religious group, 0 otherwise

Wealth index was the main explanatory variable. It is constructed using the PCA since the dataset does not contain household income or consumption or expenditure variable. (See Appendix I for the details of the computation of the wealth index). We identified the following variables that can characterize the household wealth; the household durable assets ownership that includes radio, television, refrigerator, bicycle, motorcycle, television, car; access to utilities such as electricity, having improved sanitation facility, and having improved source of drinking water [50]; and housing characteristics, such as the type of floor material. The choice of variables was based on prior literature [21, 25, 42, 48].

Mother’s age was included in our hazard function analysis. We expect that teenage mothers may lack the experience in child upbringing and this is likely to affect a child’s survival. Both mother’s and father’s education were included since parents’ education were shown to be a determinant of child survival [2, 8]. Water and sanitation are deemed essential for child health [44].^2^ Having improved source of drinking water was considered as essential for the survival of children since unimproved sources of drinking water may likely carry organisms, which could cause diarrhea, worms among others that could reduce the duration of survival. Having improved sanitary facility is an indicator of clean environment, which may also reduce the duration of survival if sanitation is poor.

At the individual level, sex of the child, birth intervals, and twin status among others were considered. For example, shorter birth interval can affect mother’s health and mother’s attention for each child will reduce. Mother’s attention may further reduce when the children are twins and this might contribute to shorter survival duration.

## Results and discussions

### Summary statistics

Table 2 shows the summary statistics of our main variables for all years under the study. The average age of a mother was about 29 years in the 1993 wave. The average birth order is 3.5 in the same wave. This means that the average mother in our dataset must have had three to four children already. However, in the 2008, the average age of a mother was 30 years while the index child may be the third child of the woman. Thus, the average age increased while the number of children decreased at this age. Even though the average number of mothers with some education increased over time, most of these mothers had only primary education. While the average number of households having improved source of water increased over time, households with improved sanitary facilities declined over time.

**Table 2:** Summary statistics of variables used for analysis

Variables	1993		1998		2003		2008	
	Mean	st-dev	Mean	st-dev	Mean	st-dev	Mean	st-dev
Duration	16.050	(10.745)	27.153	(17.837)	26.751	(17.597)	26.782	(18.088)
Household level								
Wealth status	2.971	(1.430)	2.940	(1.422)	2.934	(1.402)	2.990	(1.423)
Poorest	0.218	(0.413)	0.214	(0.410)	0.207	(0.405)	0.208	(0.406)
Poor	0.183	(0.387)	0.200	(0.400)	0.202	(0.401)	0.194	(0.395)
Middle	0.208	(0.406)	0.215	(0.411)	0.235	(0.424)	0.199	(0.399)
Richer	0.191	(0.393)	0.170	(0.376)	0.162	(0.368)	0.200	(0.400)
Richest	0.199	(0.399)	0.199	(0.399)	0.194	(0.396)	0.200	(0.400)
Mothers’ age (years)	28.651	(6.783)	30.083	(7.150)	30.498	(7.140)	30.084	(7.019)
Teenage mother	0.127	(0.333)	0.072	(0.259)	0.069	(0.254)	0.071	(0.257)
20–29	0.495	(0.500)	0.478	(0.500)	0.447	(0.497)	0.470	(0.499)
30–39	0.351	(0.477)	0.359	(0.480)	0.395	(0.489)	0.375	(0.484)
Over 40	0.084	(0.278)	0.131	(0.338)	0.124	(0.330)	0.115	(0.319)
Mothers’ education								
No education	0.397	(0.489)	0.468	(0.499)	0.475	(0.499)	0.378	(0.485)
Primary	0.547	(0.498)	0.181	(0.385)	0.214	(0.410)	0.241	(0.428)
Secondary or higher	0.055	(0.229)	0.351	(0.477)	0.311	(0.463)	0.380	(0.486)
Fathers’ education								
No education	0.367	(0.482)	0.402	(0.490)	0.459	(0.498)	0.388	(0.487)
Primary	0.463	(0.499)	0.080	(0.272)	0.083	(0.276)	0.087	(0.282)
Secondary or higher	0.170	(0.375)	0.517	(0.500)	0.457	(0.498)	0.525	(0.499)
Improved water	0.508	(0.500)	0.544	(0.498)	0.594	(0.491)	0.768	(0.422)
Improved sanitation	0.653	(0.476)	0.589	(0.492)	0.587	(0.493)	0.531	(0.499)
Individual level								
Male	0.514	(0.500)	0.492	(0.500)	0.507	(0.500)	0.510	(0.500)
Birth order	3.543	(2.254)	3.573	(2.370)	3.612	(2.341)	3.382	(2.216)
Individual level								
Preceding birth interval (months)								
Below 24	0.096	(0.294)	0.104	(0.306)	0.104	(0.305)	0.106	(0.308)
24–36	0.278	(0.448)	0.260	(0.439)	0.257	(0.437)	0.236	(0.425)
Above 36	0.420	(0.494)	0.408	(0.492)	0.418	(0.493)	0.429	(0.495)
Number of children aged ≤5	1.837	(0.962)	1.764	(0.992)	1.779	(0.941)	1.783	(0.981)
Twin	0.047	(0.211)	0.043	(0.204)	0.040	(0.196)	0.044	(0.205)
Community level								
Urban	0.279	(0.448)	0.216	(0.411)	0.271	(0.445)	0.334	(0.472)
Rural	0.721	(0.448)	0.784	(0.411)	0.729	(0.445)	0.666	(0.472)
Regional distribution								
Southern Belt	0.289	(0.453)	0.288	(0.453)	0.242	(0.429)	0.259	(0.438)
Eastern-Volta	0.216	(0.412)	0.197	(0.398)	0.153	(0.361)	0.169	(0.375)
Ashanti-Brong	0.277	(0.448)	0.204	(0.403)	0.270	(0.444)	0.236	(0.424)
Northern Belt	0.218	(0.413)	0.310	(0.463)	0.334	(0.472)	0.336	(0.472)
Religion								
No religion	0.149	(0.356)	0.099	(0.299)	0.078	(0.268)	0.050	(0.218)
Christianity	0.672	(0.470)	0.636	(0.481)	0.657	(0.475)	0.661	(0.474)
Muslim	0.127	(0.332)	0.141	(0.348)	0.207	(0.405)	0.201	(0.401)
Traditional	0.051	(0.221)	0.097	(0.296)	0.057	(0.232)	0.086	(0.280)
Others	0.002	(0.048)	0.027	(0.162)	0.001	(0.032)	0.002	(0.048)

### Rural-urban distribution of mortality among children below age 5

**Fig. 1:**
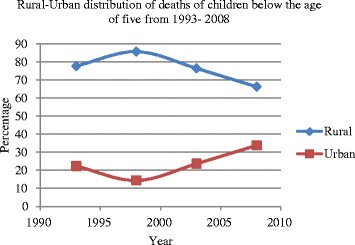
Rural-urban distribution of mortality among children below age 5 from 1993 to 2008

The number of deaths in our sample as well as under-five mortality rates^3^ are illustrated in Fig. 1. The mortality rate is measured as number of deaths per 1000 live births. Figure 1 shows the trend in the number of deaths separately for urban and rural areas. The figure indicates that under-five mortality is higher in the rural areas compared to the urban areas, but it also shows that under-five mortality in the rural areas is reducing over time while that of urban areas is increasing over the same period.^4^ This may be related to effect of urbanization and the urban poor. Studies have found that in the past decade, urbanization has increased. Although this has helped to reduce absolute poverty in the aggregate, the increase in urbanization did little for urban poverty, and children are the most affected when poor households decide to live in slums in the urban centers mainly due to poor income accessibility. Thus, poor parents are not able to afford good nutrition and better healthcare for their children [40, 46, 49].

### Distribution of wealth across regions

**Fig. 2:**
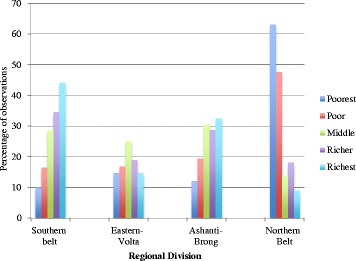
Distribution of observations within the quintiles across the region of residence

Figure 2 shows the distribution of observations (where one observation represents one child) across different levels of wealth (in quintile), separately for different regions. Sixty-three percent of children from poorest households are located in the Northern belt, and in the same region, only 9% of children are from richest households. This is the exact opposite for children who are located in the Southern belt. Forty-four percent of children in the Southern belt are from the richest household while 9% are from the poorest household.

### Kaplan-Meier

**Fig. 3:**
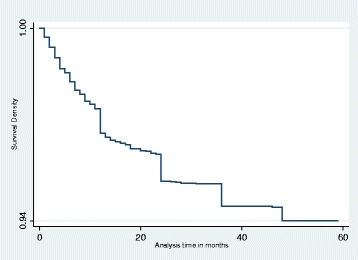
Kaplan-Meier survival estimate for under-fives

**Fig. 4:**
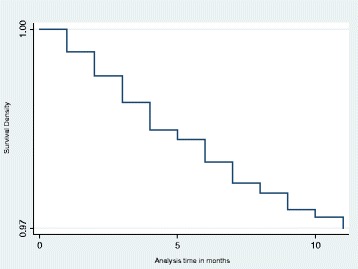
Kaplan-Meier survival estimate for infants

Our main analysis is a hazard function analysis. Before we present the results from hazard function analysis, however, it is useful to first present Kaplan-Meier (K-M) graphs. Figure 3 shows the K-M survival estimate for all children under the age of 5 years. The graph suggests that about 6% of children die before they turn 5 years. Figure 4 shows the K-M survival estimate for infants only, and it also suggests that about 3% of children die before their first birthday.

### Hazard function analysis

We used the standard Weibull hazard model with gamma frailty to estimate hazard function. We present the estimations from standard Weibull model in the Appendix in Table 6 for comparison with the standard Weibull model with gamma frailty. Before presenting the results, we briefly discuss some identification issues. The major identification problem which could lead to biased estimations and for which we are concern with is reverse causality. In the study of the effects of income on health, income can affect health and, inversely, health can affect income since one might not be able to work due to poor health, causing a reverse causality problem. However, the main subjects of this study are children below the age of 5 years. These children are less likely to contribute directly to the wealth of the household. Therefore, reverse causality may be considered to be much less of a problem in this study.

In addition to the fact that our subjects are children below age 5, some studies, such as that of Acemoglu and Johnson [1] showed in their study of the effect of life expectancy on economic growth that there was no evidence that increase in life expectancy which was mainly driven by child mortality, led to a faster growth of income per capita or output per worker. Thus, reverse causality does not substantially bias our estimate. Two other identification problems, which could lead to biased estimations, are how to account for unobserved heterogeneity and dependence^5^ among observations. To account for these, we include a gamma frailty term in our model^6^ [4, 23, 31, 36]. Thus, the hazard function becomes a function of both the observed covariates and unobserved frailties associated with the individual.

### Wealth effect

**Fig. 5:**
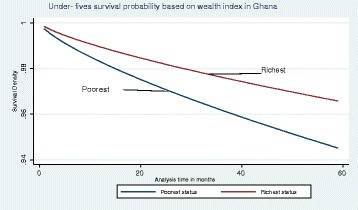
Graph of Weibull survival rate by poorest and richest classes for under-fives

Now, we turn to our results. Model 1 in Table 3 shows the estimates of effect of household wealth on the survival of all children under the age 5. Household wealth status has a negative and significant effect on child survival. Thus, a child is more likely to survive when he/she is from a household with high wealth status. To understand the magnitude of the wealth effects more clearly, we computed the survival probability for the top and the bottom wealth quintiles, while holding others factors constant. Figure 5 shows the results, which suggests that the top wealth quantile households had about 3.5% child mortality while bottom quantile had child mortality of 5.5%. So the difference is 2%, which is relatively high. Thus, the survival probability is lower for poorest but relatively high for the richest.

**Table 3:** Effect of wealth and other factors on risk of death among children in Ghana-Estimated with Gamma Frailty

Variables	All under 5			Infants
	Model 1	Model 2	Model 3	Model 4
	Coefficient (s. e.)			
Household level				
Wealth status	*−0.116* ^a^		−0.272^a^	*−0.095*
	*(0.054)*		(0.111)	*(0.072)*
Poorest		*0.523* ^a^		
		*(0.234)*		
Poor		*0.405* ^b^		
		*(0.212)*		
Middle		*0.298*		
		*(0.195)*		
Richer		*0.245*		
		*(0.189)*		
Mothers’ age (years)				
20–29	0.241	0.246	0.238	0.197
	(0.289)	(0.289)	(0.289)	(0.358)
30–39	0.087	0.093	0.070	0.126
	(0.315)	(0.316)	(0.316)	(0.399)
Over 40	−0.278	−0.269	−0.305	−0.389
	(0.389)	(0.370)	(0.371)	(0.485)
Mothers’ education				
Primary	0.0001	−0.002	0.009	0.128
	(0.139)	(0.139)	(0.139)	(0.889)
Secondary or higher	−0.437^c^	−0.432^c^	−0.457^c^	−0.118
	(0.163)	(0.163)	(0.164)	(0.216)
Fathers’ education				
Primary	−0.195	−0.195	−0.184	−0.509^a^
	(0.173)	(0.173)	(0.174)	(0.248)
Secondary or higher	−0.405^c^	−0.403^c^	−0.410^c^	−0.367^b^
	(0.137)	(0.137)	(0.137)	(0.183)
Safe water	0.012	0.013	0.048	0.024
	(0.116)	(0.119)	(0.119)	(0.157)
Improved sanitation	0.187	0.186	0.144	0.082
	(0.146)	(0.149)	(0.147)	(0.191)
Individual level				
Male	−0.025	−0.025	−0.028	0.016
	(0.099)	(0.099)	(0.099)	(0.133)
Birth order	0.073^a^	0.072^a^	0.076^a^	0.065
	(0.033)	(0.033)	(0.033)	(0.046)
Preceding birth interval (months)				
Below 24	0.517^c^	0.514^c^	0.524^c^	0.711^c^
	(0.164)	(0.164)	(0.165)	(0.208)
Above 36	−0.499^c^	−0.501^c^	−0.502^c^	−0.493^c^
	(0.120)	(0.120)	(0.121)	(0.167)
Number of children aged ≤5	−1.268^c^	−1.266^c^	−1.270^c^	−1.042^c^
	(0.075)	(0.075)	(0.075)	(0.098)
Twin	1.90^c^	1.810^c^	1.899^c^	2.047^c^
	(0.299)	(0.229)	(0.231)	(0.308)
Community level				
Urban	−0.126	−0.114	−0.0141	−0.206
	(0.143)	(0.147)	(0.145)	(0.194)
Regional distribution				
Southern Belt	−0.357^c^	−0.362^a^	−0.350^a^	−0.417^b^
	(0.170)	(0.170)	(0.170)	(0.230)
Ashanti-Brong	−0.496^c^	−0.500^c^	−0.486^c^	−0.598^a^
	(0.179)	(0.179)	(0.180)	(0.244)
Eastern-Volta	−0.599^c^	−0.606^c^	−0.585^c^	−0.587^a^
	(0.197)	(0.199)	(0.198)	(0.259)
Religion				
No religion (excluded category)				
Christianity	−0.167	−0.169	−0.131	−0.122
	(0.189)	(0.189)	(0.190)	(0.264)
Muslim	−0.577^b^	−0.584^b^	−0.559^b^	−0.357
	(0.297)	(0.298)	(0.299)	(0.382)
Traditional	−0.221	−0.222	−0.217	0.041
	(0.219)	(0.220)	(0.221)	(0.300)
Others	−0.098	−0.100	−0.063	−0.037
	(0.193)	(0.193)	(0.195)	(0.272)
Community level				
Period				
Year 1998	0.338^b^	0.334^b^	−0.016	0.219
	(0.195)	(0.195)	(0.352)	(0.247)
Year 2003	0.126	0.123	−0.350	−0.122
	(0.195)	(0.195)	(0.374)	(0.250)
Year 2008	0.03	0.023	−0.721^b^	−0.026
	(0.206)	(0.206)	(0.412)	(0.261)
(Wealth) × (year 1993)			−*0.272* ^a^	
			*(0.111)*	
(Wealth) × (year 1998)			*0.135*	
			*(0.121)*	
(Wealth) × (year 2003)			*0.181*	
			*(0.126)*	
(Wealth) × (year 2008)			*0.277* ^a^	
			*(0.135)*	
Log *α* (shape parameter)	−0.226^c^	−0.227^c^	−0.224^a^	0.095
	(0.050)	(0.05)	(0.051)	(0.072)
Log likelihood	−2290	−2290	−2287	−1388
Prob > chi-square	0.000	0.000	0.000	0.000
Theta, *θ*	1.305^a^	1.310^a^	1.374^a^	3.001^b^
	(0.669)	(0.669)	(0.695)	(2.427)
Prob > chi-square for *θ*	0.005	0.004	0.004	0.057

^a^Indicates significance at 5%

^b^Indicates significance at 10% level

^c^Indicates 1% significance level

An upward move into the next highest class in wealth quintile by a household reduced the risk of child death by a multiplicative factor of 89%. Before reaching their fifth birthday, the risk of dying if a child is from the poorest household was about two times higher than one of the same age from richest household. This could be an indication that high under-fives’ mortality rates experienced over the years have its sources rooted in the circumstances of the poorest/poor households. However, we found that such disparity in survival rates by wealth status gradually reduced overtime. The findings of significant wealth effect on child mortality are consistent with of other studies [10, 11, 13, 14, 18]. What then could be the source of these wealth effects in the Ghanaian situation?

Many reasons may account for the high risk seen among children in the poorest/poor households; thus, household, health systems, and program level mediators could account for this. For example, poor households may not afford to provide basic needs of the children; they are unable to pay for extra medical bills aside what the National Health Insurance Scheme provides; and there could also be unequal access to health services, low human and material resources in facilities that serve the poor, low or sometimes the lack of technical quality of health care for the poor, and universality nature of programs which should alleviate poverty. In Ghana, there is a qualitatively significant difference between the rich and the poor. The rich are able to provide at least the basic needs of their households including nutritious food, safe water, enough sleeping rooms, safe environment, and also pay extra medical bills among others. These basic needs are not met for poorest/poor households. Thus, children from low-income families are more likely to be subject to more health shocks [18].

**Fig. 6:**
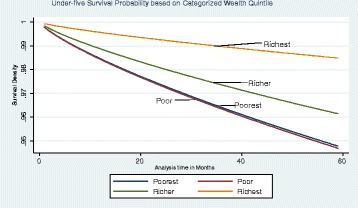
Graph of survival probabilities based on model 2

Model 2 controls for wealth as categorical dummies to capture possible non-linear effect, where wealth index is divided into wealth quintile dummies. In column 2, we found that the hazard of death was twice for a child from the poorest household compared to a child from a richest household. Holding all other factors constant, we computed the survival probability for all the quintiles. This is shown in Fig. 6, which suggests that the richest class would have child mortality of 1.5% while the poorest has 5.5% by the 59th month; so the difference is 4%. From the graph, the survival probability for the poorest and the poor were almost the same and so is the difference between the richest and the poor. The difference is relatively higher compared to the earlier estimation that considered the coefficient of wealth status to be constant for all categories. The difference may be attributed to reasons as already discussed.

Model 3 examines if wealth effects have changed over time. In the past 20 years, Ghana has considerably improved its provision of reproductive and child health services. If the public health service were a substitute for household wealth, we would expect that wealth effect must decline over time. Thus, model 3 includes interaction between wealth and year dummies to estimate this effect. As shown in Table 3, the effect of wealth in 1993 is negative and significant. However, the interactive coefficients are all positive and monotonically increasing over time, and therefore, the wealth effects becomes gradually less negative over time. In fact, in 1998, it was close to zero. Thus, the effect of wealth reduced over time. This is consistent with our expectation. The trend may be attributed to gradual strengthening of public health systems to support child health care over the years. For example, vaccination trend has increased from 55% in 1993 to 79% in 2008; household bed net use increased from 4% in 2003 to 39% in 2008, and between 1993 and 2008, health facilities including Community-Based Health Planning and Services (CHPS) compounds increase by about 30% across Ghana^7^ and National Health Insurance Scheme was introduced in 2003. However, the result of this study indicates that these efforts by the Government will not be enough to improve under-fives’ survival if it is not complemented with an increase in household wealth.

## Other determinants of child mortality

Other variables, which are also of interest, are discussed below using the results mainly from Model 1. First, the risk of childhood mortality was significantly high for children born less than 2 years after a previous sibling whiles it was significantly low for children born more than 3 years after a previous sibling. A child born less than 2 years after a previous sibling was 1.7 [Exp (0.517) = 1.7] times more likely to die whiles the risk reduced by a multiplicative factor of 61% among children born more than 3 years after a previous sibling. This may be due to many reasons; common among these are (1) competition for parents’ limited time and resources, (2) the inability to allot enough time for a child if his/her birth was earlier than desired, and (3) most importantly, the transmission of diseases among closely spaced siblings [19]. Our results reaffirmed the importance of child spacing.

Furthermore, children born to mothers who had at least secondary education had their risk of death reduced. This finding is consistent with Blunch’s [3] finding on rural Ghana. Father’s level of education was also highly statistically significant. Children born to fathers who have at least secondary education have their risk of death reduced. Whereas we find both parents education almost equally counted in determining child mortality, some studies (see for example, Chalasani and Rutstein [14], Chalasani [13], Caldwell [8]) found that mothers’ education had a relatively higher impact on child mortality than fathers’ education and any other socioeconomic factors. Breierova and Dufflo [7] in their program evaluation in Indonesia similarly found that increase in both parents’ education had a strong causal effect on the reduction of child mortality. The trend may be due to the changing socialization circumstances in Ghana where men have increasingly become more concern about child care; and it may further be due to the current nature of ante-natal health education which is gradually involving husbands of pregnant women.

Childhood risk of death reduced by a multiplicative factor of about 28% [Exp (−1.268) = 28%] when the number of children who were less than 5 years in a household reduced by one. Also, if the index child is a twin, hazard of death would increase by about seven times compared to a child of single birth. The risk of children who were twin may be attributed to the same reasons as found in literature for birth intervals. However, the risk is seen to be very high for the twin child due to the fact that competition for parents’ time occurs at the same time period and so handling twins becomes challenging for parents.

Safe water and improved sanitation did not have significant coefficients. Although a recent study by Ezeh et al [20] in Nigeria found that the probability of childhood mortality significantly high among children who have lack of access to safe water and improved sanitation, the reason for insignificant coefficients for “safe water” and “improved sanitation” in our study is perhaps due to the fact that those variables were used to construct wealth index. Thus, it could be that the effects of these variables were mostly captured by the wealth status. Also, urban dummy variable had negative but insignificant coefficient. This may appear contradict Fig. 1 that shows that urban areas generally had lower mortality rate throughout our sample period. The insignificant estimate may be due to the fact that most of the urban areas are concentrated in the Southern belt and Ashanti-Brong regions. Thus, the regional dummies especially southern belt dummy may mostly capture the effect of urban area. Even though it had weak significance; children born to Muslim households were less likely to die before reaching their fifth birthday than those born into households who had no religion.

Furthermore, the risk of childhood mortality significantly reduced in relations to regional location of the household. The risk of dying for children born in households located in the Southern belt, Ashanti-Brong and Eastern-Volta reduced by a multiplicative factor of 70, 61, and 55%, respectively, compared to those born within households in the Northern belt. Thus, a child faces a high hazard of death when he/she is located in a household in the Northern Belt. This may be due to poor income and geographical access, which directly affects the health of children [49]. The findings on regional location using child survival as a major indicator of household’s economic status and by extension, the economic development of the region, are supported by findings by Overseas Development Institute and Centre for Policy Analysis [37] of Ghana. They indicated that the three northern regions of Ghana, which are captured as Northern belt in this study, are persistently the poorest; and unfortunately, the stable economic growth, which has been experienced in Ghana since the early 1990s has not extended to the north. Generally, the risk of child mortality reduced over the historic period under the study. The shape of the hazard rate *α* is 0.59, which is less than 1, indicating that there is negative duration dependence. Thus, if children were alive for a longer period, they were less likely to die.

### Robustness check: infant survival

**Fig. 7:**
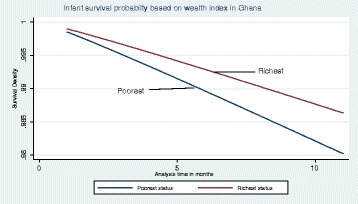
Graph of Weibull hazard rate by poorest and richest classes for infants

As shown in Table 3, model 4 shows a model restricted to the duration 0–11 months, as a robustness check. As can be seen, the sign of coefficients were unaltered, though the main explanatory variable was not statistically significant. The difference in survival between infants from poorest and richest households is illustrated in Fig. 7, which shows that the poorest are less likely to survive compared to the richest over the same period. The insignificant estimate suggests that wealth status of the household is not a major determinant survival in infancy. The results is not out of place since it is theoretically known that at the early stages of life, biological and genetic factors mediate more in mortality, and income effect is expected to be stronger after infancy.

## Conclusions

Empirical evidence of the consequence of households’ wealth status on child survival is scarce in developing countries, particularly in Sub-Saharan Africa. We use four waves of data from DHS for the historic years of 1993–2008 to study a policy-relevant question, which has not been studied with Ghanaian data. Thus, we estimate the effect of wealth on child survival in Ghana, and our study unravels the relationship between child health and many economic and social factors.

We found that household wealth status had a significant effect on child survival. Results from this study as well as other studies over the years have provided evidence that the risk of child mortality is highest among the poor [14]; therefore, there is an increasing need to appropriately target the poor. This could be done by making services more accessible to the poor, increase the availability of human and material resources in facilities that serve the poor; make available and increase technical quality of health care services to the poor (see, for example, literature review by [49]); and implementing policies which alleviate poverty and sustain wealth in deprived areas targeting such disadvantaged groups. However, the cost-effectiveness of such policy strategies is beyond this study and is recommended for future research. The study further recommends that as a developing country, Ghana needs to conduct studies that will help it appropriately target the poor before implementing the various pro-poor programs.

Other than wealth effect, we found the following results, which should also be emphasized. Estimates of this study suggest that preceding birth interval which is commonly known as “child spacing” had a significant effect on both infant and child survival. These finding are similar with studies by Rutstein [41] and DaVanzo et al. [19]. Based on our findings, we recommend that policy makers should make it as part of their message when educating parents about family planning to wait at least 24 months after birth to conceive the next child in order to reduce the risk of death among children below the age of 5. Common approaches to prolong child spacing are through the use of family planning methods and also effective parental education.

Another important finding of this study is the high risk of death in childhood when the index child is a twin. This may have similar reasons as those of child spacing, but in addition, competition for parent’s limited time occurs at the same time and this is a formidable challenge for parents. This finding is similar to that of Uthman et al. [47] and Hong [26]. The evidence suggests that it is important to have a considerable number of screening programs at the community level in order to identify high-risk pregnancies and to refer them appropriately in order to reduce the risk. It is also important that once such high-risk pregnancies are identified, the parents are given enough education on how to handle the children when they are born.

We found that an increase in both maternal and paternal education reduced the risk of death especially among children. This may be because educated parents become more capable to take steps to protect their children from diseases. Findings are similar to those of Breierova and Dufflo [7]. Thus, educating both females and males is essential for child survival in Ghana.

Furthermore, we found the survival in all children below age 5 years vary with the region of residence, when other variables are held constant. As already shown in the results section, children in the Northern belt had the highest risk of death. This is not to underscore the risk of deaths in households in the other regions; however, this does suggest that it is only necessary that poverty reduction and wealth sustenance initiatives targeted the deprived regions, reduce and if possible totally mute regional disparities in order to improve the wealth status of households and, in so doing, reduce the risk of dying among children below the age of 5 years in Ghana.

Although in this study, we try to produce unbiased estimates, it is important to notice that household wealth, which serves as a proxy for the economic status of the household was determined, based on household assets and characteristics indicated by the head of the household during the period of the survey. Any over-reporting or under-reporting of the quantity of assets will likely introduce some degree of measurement errors in a household wealth status estimations; although thorough evaluation of DHS data has shown that the data are reasonably well reported.

Furthermore, the conclusions made in this study were based on the analysis of only one country data and so generalizing findings for developing countries should be done with caution.

## Appendix I

### Wealth index construction

We develop a proxy for the household economic status using the PCA. Research has shown that the use of PCA in the construction of the wealth index based on household assets and housing characteristics is robust, valid, and correctly represents the long-run household economic status [13, 14, 21, 25, 41, 47]. PCA is a multivariate technique used to extract from a set of variables the few orthogonal linear combinations of the variables that capture the common information most successfully. In this, a number of variables in the data set are reduced into a smaller number of dimensions. First, the asset variables used are changed into indicator variables, which are separately entered in a linear multivariate regression equation that will create weights on the variables; and so each principal component is a weighted linear combination of the original variable. From the set of correlated variables, the PCA extracts a set of uncorrelated principal components.

Supposed that there are *n* correlated variables, *X*_1_-*X*_*n*_ representing the number of assets in each household *i*, each variable is normalized by using its own mean and standard deviation.

*X*_1_ = (*x*_1_ − *x*_1_*)/*S*_1_*, where *x*_1_* is the mean of all values of the first variable and *S*_1_* is its standard deviation. Given a set of variables from *X*_1_ through to *Xn*, the principal components are expressed as:

$$ {\mathrm{PC}}_1 = {\alpha}_{11}{X}_1 + {\alpha}_{12}{X}_2 + \dots + {\alpha}_{1n}{X}_n $$

$$ {\mathrm{PC}}_m = {\alpha}_{m1}{X}_1 + {\alpha}_{m2}{X}_2 + \dots + {\alpha}_{mn}{X}_n $$

where *α*_*mn*_ is the coefficient or weight or the factor score for the *m*th principal component and the *nth* variable.

When PCA is used, the variance for each principal component (PC) is given by the eigenvalue of the corresponding eigenvector. Each principal component is the sum of each variable multiplied by its weight; weight is different for each variable in each principal component and is effectively defined by a factor score. The components are ordered such that the first principal component (PC_1_) explains the largest part of variation in the original data and corresponds to the largest eigenvalue of the correlation matrix of *X*, subject to the constraint that the sum of the squared weights is equal to one (*a*_11_^2^ + *a*_12_^2^ + … + *a*_1*n*_^2^). PC_1_ is uncorrelated to the second component and the other components, which give additional variations; and PC_1_ is assumed to represent the economic status.

It is important to note that the number of households in each wealth group is based on the factor scores obtained from the principal component analysis. Higher positive scores are assigned to variables that are more likely to be associated with the richer households while the negative scores are to those variables that are more likely to be associated with the poorer households. The higher the resulting score, the higher the contribution of that variable to the wealth index. Appendix Table 4 shows the principal components and Table 5 shows the scoring coefficients constrained on the fact that the sum of squares is equals to 1.

## Appendix 2

**Table 4:** Principal components/correlation for 1993, 1998, 2003, and 2008

Components	Eigenvalues			
	1993	1998	2003	2008
1	3.222	3.171	3.279	3.086
2	1.819	1.968	1.793	1.803
3	1.421	1.323	1.450	1.511
4	1.000	.985	.992	1.022
5	.895	.934	.910	.924
6	.857	.873	.828	.823
7	.725	.704	.787	.790
8	.663	.702	.688	.698
9	.582	.530	.479	.582
10	.434	.452	.447	.447
11	.383	.355	.348	.316
12	.002	.003	0	0

**Table 5:** Scoring coefficients of standardized variables constraint: sum of squares (column loading) = 1

Variables	1993	1998	2003	2008
Radio	.275	.268	.176	.157
Television	.431	.448	.440	.466
Refrigerator	.436	.430	.433	.415
Bicycle	−.047	−.135	−.169	−.175
Motorcycle	.084	.083	.026	.017
Car	.255	.221	.253	.191
Electricity	.418	.430	.417	.460
Safe water	.240	.240	.216	.085
Improved toilet facility	.068	.176	.254	.346
Cement/tile floor	−.212	−.032	−.170	.047
Wood type floor	.420	.367	.393	.291
Earth/mud floor	−.126	−.242	−.162	−.305

**Table 6:** Effect of wealth and other factors on child survival in Ghana-Estimated with Standard Weibull

Variables	All under-fives			Infants
	Model 1	Model 2	Model 3	Model 4
		Coefficient (s. e.)		
Household level				
Wealth status	*−0.120* ^a^		−0.257^a^	*−0.096*
	*(0.050)*		(0.105)	*(0.067)*
Poorest		*0.523* ^a^		
		*(0.234)*		
Poor		*0.405* ^a^		
		*(0.212)*		
Middle		*0.298*		
		*(0.195)*		
Richer		*0.245*		
		*(0.189)*		
Mothers’ age (years)				
20–29	0.225	0.232	0.217	0.226
	(0.272)	(0.273)	(0.272)	(0.336)
30–39	0.136	0.147	0.119	0.197
	(0.295)	(0.296)	(0.295)	(0.369)
Over 40	−0.178	−0.164	−0.201	−0.183
	(0.344)	(0.345)	(0.345)	(0.446)
Mothers’ education				
Primary	(0.0030)	−0.002	0.004	0.155
	(0.127)	(0.127)	(0.127)	(0.173)
Secondary or higher	−0.384^a^	−0.377^a^	−0.399^b^	−0.094
	(0.152)	(0.152)	(0.152)	(0.201)
Fathers’ education				
Primary	−0.148	−0.149	−0.134	−0.453^c^
	(0.160)	(0.160)	(0.160)	(0.229)
Secondary or higher	−0.407^b^	−0.407^b^	−0.0409^b^	−0.374^a^
	(0.127)	(0.127)	(0.127)	(0.169)
Safe water	−0.004	−0.009	0.028	0.007
	(0.107)	(0.110)	(0.110)	(0.144)
Improved sanitation	0.256^c^	0.254^c^	0.128	0.128
	(0.133)	(0.135)	(0.134)	(0.177)
Individual level				
Male	−0.039	−0.039	−0.042	0.023
	(0.091)	(0.091)	(0.091)	(0.123)
Birth order	0.059^c^	0.058^c^	0.061^a^	0.049
	(0.030)	(0.030)	0.030	(0.041)
Preceding birth interval (months)				
Below 24	0.361^a^	0.357^a^	0.362^a^	0.607^b^
	(0.141)	(0.141)	(0.141)	(0.180)
Above 36	−0.448^b^	−0.451^b^	−0.449^b^	−0.429^b^
	(0.110)	(0.110)	(0.111)	(0.150)
No. of children aged ≤5	−1.185^b^	−1.183^b^	−1.185^b^	−0.973^b^
	(0.064)	(0.064)	(0.064)	(0.082)
Twin	1.571^b^	1.571^b^	1.566^b^	1.300^b^
	(0.175)	(0.175)	(0.175)	(0.211)
Community level				
Urban	−0.074	−0.060	−0.083	−0.153
	(0.134)	(0.138)	(0.134)	(0.180)
Regional distribution				
Southern Belt	−0.391^a^	−0.398^a^	−0.382^a^	−0.426^a^
	(0.155)	(0.156)	(0.155)	(0.209)
Ashanti-Brong	−0.514^b^	−0.516^b^	−0.497^b^	−0.595^b^
	(0.166)	(0.166)	(0.166)	(0.228)
Eastern-Volta	−0.632^b^	−0.641^b^	−0.618^b^	−0.59^a^
	(0.183)	(0.185)	(0.183)	(0.241)
Religion				
Christianity	−0.203	−0.205	–0.176	–0.123
	(0.173)	(0.173)	(0.174)	(0.244)
Muslim	–0.567^a^	–0.569^a^	–0.557^c^	–0.325
	(0.280)	(0.280)	(0.281)	(0.358)
Traditional	–0.208	–.0208	–0.207	0.057
	(0.203)	(0.203)	(0.204)	(0.278)
Others	–0.068	–0.069	–0.041	0.001
	(0.177)	(0.177)	(0.177)	(0.270)
Community level				
Period				
Year 1998	0.254	0.249	–0.039	0.195
	(0.181)	(0.181)	(0.337)	(0.228)
Year 2003	0.093	0.087	–0.309	–0.142
	(0.183)	(0183)	(0.348)	(0.233)
Year 2008	0.021	–0.027	–0.682	–0.066
	(0.194)	(0.194)	(0.382)	(0.242)
(Wealth) × (year 1993)			*–0.257* ^a^	
			*(0.105)*	
(Wealth) × (year 1998)			*0.111*	
			*(0.114)*	
(Wealth) × (year 2003)			*0.155*	
			*(0.118)*	
(Wealth) × (year 2008)			*0.247* ^*a*^	
			*(0.126)*	
Log *α* (shape parameter)	–0.292^b^	–0.292^b^	–0.292^b^	0.039
	(0.042)	(0.042)	(0.042)	(0.059)
Log likelihood	–2293	–2293	–2291	–1389
Prob > chi-square	0.000	0.000	0.000	0.000

^a^Indicates significance at 5%

^b^Indicates 1% significance level

^c^Indicates significance at 10% level

## Abbreviations

CHPS: Community-Based Health Planning and Services
DHS: Demographic and Health Survey
K-M: Kaplan-Meier
MDGs: Millennium Development Goals
PCA: Principal Component Analysis
UNICEF: United Nations International Children’s Emergency Fund
WHO: World Health Organization

## Footnotes

1 Box-Steffensmeier and Jones [4] described this using political science data. Similar model was used by Hong et al. [27]. Choe [15] also used it to study infant and childhood mortality. Model is further explained by Lancaster [33]. Also refer to Gutierrez [24] on how to handle survey for survival analysis.

2 Safe source of drinking water is defined as private or public piped water, borehole/tube well, protected dug well/spring, and rain collection, and improved sanitary facility is defined as having a flush or pours flush into pipe sewer system, septic tank or pit latrine, and ventilated improved latrine (WHO/UNICEF 2009).

3 Data source for mortality rates is from Ghana Demographic and Health Survey report (2008).

4 The percentage of deaths in the rural area was 77.7, 85.7, 76.4, and 66.2% in 1993, 1998, 2003, and 2008, respectively, while in the urban areas, it was 22.3, 14.3, 23.6, and 33.8% for the same years.

5 An important assumption of the hazard model is that the observations are independent. However, data on children are collected from mothers who may have more than one child; therefore, children may not be independent observations. Refer to Klein and Moeschberger (1997) for detailed explanation.

6 Model described by Box-Steffensmeier and Jones [4] similar model used by Box-Steffensmeier, Linn & Smidt [5]. Model also proposed by Hougaard [28].

7 Ghana Statistical Service (GSS) [22]: Multiple indicator cluster survey (MICS).
